# Effect of Denosumab on Bone Mineral Density of Hematopoietic Stem Cell Transplantation Recipients

**DOI:** 10.1155/2020/3410921

**Published:** 2020-05-04

**Authors:** Chaiho Jeong, Hee-Je Kim, Seok Lee, Moo Il Kang, Jeonghoon Ha

**Affiliations:** ^1^Division of Endocrinology and Metabolism, Department of Internal Medicine, Seoul St. Mary's Hospital, College of Medicine, The Catholic University of Korea, Seoul, Republic of Korea; ^2^Division of Hematology, Department of Internal Medicine, Seoul St. Mary's Hospital, College of Medicine, The Catholic University of Korea, Seoul, Republic of Korea

## Abstract

**Purpose:**

Denosumab is a monoclonal antibody that prevents the development of osteoclasts. The effect of denosumab in solid organ transplant recipients has been elucidated, but its effect in haematopoietic stem cell transplantation recipients has not been studied yet. The aim of this study was to determine the effectiveness and safety of denosumab in haematopoietic stem cell transplantation recipients.

**Methods:**

We retrospectively evaluated 33 female patients with osteoporosis (mean age 52.6 ± 9.8 years) following allogeneic haematopoietic stem cell transplantation. Patients were treated with denosumab every 6 months for 12 months. Changes in bone mineral density were evaluated for denosumab-treated patients in a 12-month interval after the first administration of denosumab.

**Results:**

Significant increases in bone mineral density were observed in all measured skeletal sites including 4.39 ± 6.63% in the lumbar spine (*p*=0.014), 3.11 ± 7.69% in the femoral neck (*p*=0.048), and 1.97 ± 6.01% in the total hip (*p*=0.138). The bone turnover marker serum cross-linked C-terminal telopeptide of type 1 collagen was decreased at 18 months (−51.6 ± 17.6%, *p* < 0.001). No serious symptomatic hypocalcaemia was observed. Serious adverse drug reactions requiring drug discontinuation were not observed.

**Conclusion:**

Denosumab improved bone mineral density in haematopoietic stem cell transplantation recipients. The use of denosumab could be a good therapeutic option without causing severe adverse effects in recipients of haematopoietic transplantation.

## 1. Introduction

Osteoporosis is a serious disease that affects more than 200 million people worldwide [1]. Its incidence is escalating with an increase in the population of elderly. Osteoporosis leads to decreased bone strength and consequent increase in the risk of fracture, leading to considerable morbidity and decline in the quality of life [2, 3]. To date, a variety of agents have been approved to treat osteoporosis. Several antiresorptive agents such as bisphosphonates (BPs), selective oestrogen receptor modulators, and denosumab have successfully decreased the incidence of new fractures by 30–50% [4, 5]. Particularly, denosumab, the first approved biologic agent for the treatment of osteoporosis, is a powerful antiresorptive drug that significantly reduces the risk of hip, vertebral, and nonvertebral fractures in patients with postmenopausal osteoporosis [6]. Clinical guidelines have recommended denosumab as the first-line treatment for patients having osteoporosis without fracture and for those having severe osteoporosis with fracture [7, 8]. Recently, advances in transplantation techniques and supportive care have led to an increase in the long-term survival following haematopoietic stem cell transplantation (HSCT), which is the treatment of choice for some malignant haematological diseases [9]. Reportedly, the incidence of osteopenia at 4–6 years after HSCT in adults is nearly 50%, and the incidence of osteoporosis at 2 years after HSCT is nearly 20% [10]. Bone loss and consequent bone fracture lead to morbidity in HSCT patients. With an increase in the long-term survival of HSCT patients, osteoporotic fracture is becoming an increasingly serious problem among these patients. BPs are the most frequently studied drugs for the HSCT-associated loss of bone mineral density (BMD). In previous studies, BPs have shown an increase in BMD in the early post-HSCT period and during their continued use [11–13]. However, the effect of denosumab on BMD after transplantation has not been clearly verified yet. Particularly, no study has reported the efficacy of this drug in HSCT-induced bone loss. Thus, the aim of the present study was to determine the effectiveness and safety of denosumab in HSCT recipients.

## 2. Patients and Methods

**Figure 1:**
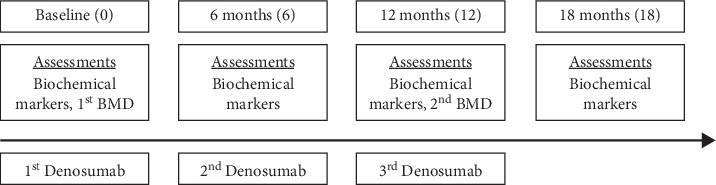
Assessments flow of the study.

We retrospectively evaluated 33 postmenopausal patients with osteoporosis following allogeneic HSCT. Patients with multiple myeloma were excluded because multiple myeloma can invade the bone easily, rendering BMD value unreliable. The period since transplantations was less than 3 years in all patients upon beginning denosumab. Patients were drug naïve patients who have not previously been treated for osteoporosis. Patients were treated with denosumab (60 mg, S.C.) three times every 6 months between 2017 and 2019 in a single tertiary center. All patients received daily elemental calcium (500 mg) as calcium carbonate with cholecalciferol (1000IU). The BMD of the lumbar spine (lumbar vertebra L1-4) and the BMD of the femur neck and total hip were measured by dual energy X-ray absorptiometry using Hologic Delphi W (Hologic Inc., Bedford, MA). The coefficient of variation was determined to be 1.2% at the lumbar spine and 1.9% at the femoral neck. Denosumab-treated patients were evaluated using DEXA at baseline and 12 months after the first administration of denosumab. Blood samples were collected after overnight fasting. Biochemical tests including serum cross-linked C-terminal telopeptide of type 1 collagen **(**CTX), serum 25(OH) vitamin *D*, and serum calcium level were performed every 6-month after denosumab treatment (0, 6, 12, and 18 months). During the 18-month study period, BMD was measured twice (0 and 12 months), and denosumab was administered three times (0, 6, and 12 months). Biochemical markers were measured four times (0, 6, 12, and 18 months) (Figure 1). Normal level of CTX is < 0.573 ng/mL in the premenopausal state and <1.008 ng/mL in the postmenopausal state. Serum calcium was corrected for changes in serum albumin concentration. The study was approved by the Institutional Review Board of Seoul St. Mary's Hospital, the Catholic University of Korea (KC19RCSI0731).

## 3. Statistical Analysis

Continuous variables were expressed as the mean ± standard deviation or percentage unless otherwise stated. Categorical variables were described based on relative frequencies. For comparison, the paired t-test was used for continuous variables with normal distribution. The Wilcoxon signed-rank test was used to evaluate the differences of variables when their values were not normally distributed. A two-tailed *p* value of less than 0.05 was considered statistically significant. All statistical analyses were performed using IBM SPSS Statistics for Windows v24.0 (IBM Corp., Armonk, NY, USA).

## 4. Results

Baseline characteristics of patients before denosumab treatment are summarised in Table 1. The mean age of these female patients was 52.6 ± 9.8 years. Baseline 25-hydroxyvitamin *D* level was 30.3 ± 10.0 ng/mL. All patients were treated with immunosuppressive agents, especially calcineurin inhibitors, and high doses of intravenous steroids during peri-HSCT and post-HSCT periods to prevent and treat graft versus host disease (GVHD). The accumulation doses of steroids reached up to 6.19 ± 6.48 mg/kg for intravenous dexamethasone and 20.44 ± 17.02 mg/kg for intravenous methylprednisolone. Twenty-two patients (66.7%) continued to receive steroids during denosumab treatment.

**Figure 2:**
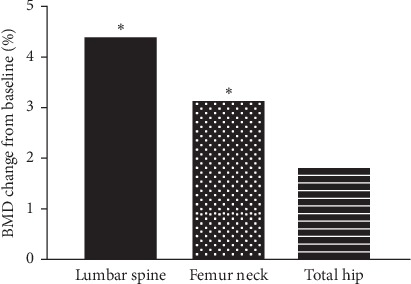
The percentage changes in BMD at 12 months after treatment with denosumab. ^*∗*^*p* < 0.05 compared to baseline.

After 12 months of denosumab treatment, significant increases in BMD were observed in all evaluated skeletal sites including 4.39 ± 6.63% in the lumbar spine (*p*=0.014), 3.11 ± 7.69% in the femoral neck (*p*=0.048), and 1.97 ± 6.01% in the total hip (*p*=0.138) (Figure 2).

**Figure 3:**
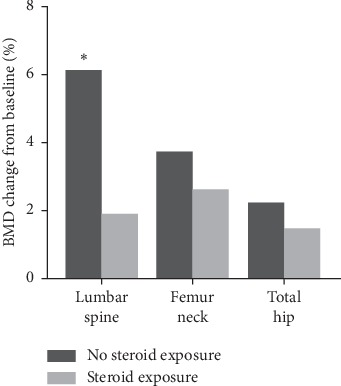
The percentage changes in BMD at 12 months after treatment with denosumab according to steroid exposure during the denosumab treatment. ^*∗*^*p* < 0.05 between the groups.

Patients who were receiving steroids during denosumab treatment did not respond to the denosumab therapy when compared with patients who did not receive steroids during denosumab treatment. Especially, the lumbar spine BMD of steroid recipients during the denosumab therapy increased only by 1.91 ± 6.77% when compared with that of patients who did not receive steroids (increase by 6.12 ± 5.96%). The difference in BMD increase between these two sets of patients was statistically significant (*p*=0.041) (Figure 3).

The bone turnover marker cross-linked C-terminal telopeptide of type 1 collagen was decreased significantly at 18 months (−51.6 ± 17.6%, *p* < 0.001) (Table 2). No serious symptomatic hypocalcaemia (8 mg/dL) was observed. Serious adverse drug reactions requiring drug discontinuation were not observed. However, during the use of denosumab, 2 (6.1%) patients were hospitalised due to infection. One patient had herpes zoster and the other experienced sinusitis.

## 5. Discussion

In the present retrospective study, we analysed the results of denosumab therapy in patients after HSCT. Denosumab induced a significant increase in BMD in HSCT patients. The increase in the lumbar spine BMD was substantial despite previous steroid and immunosuppressant therapy.

Osteoporosis after transplantation is multifactorial. It occurs through a complex interaction of pre-HSCT, peri-HSCT, and post-HSCT factors [14]. Its pathogenesis involves altered bone metabolism, immunosuppressive therapy, corticosteroid treatment in the peritransplant period, and vitamin *D* deficiency [15]. We have previously confirmed that differentiation of bone marrow stromal cells into osteoblasts is impaired after HSCT, which might contribute to post-HSCT bone loss [16]. Furthermore, premature menopause in women and decreased levels of androgens in men are also the major causes of transplant-related bone loss [17]. In addition, it has been suggested that the increased level of interleukin-6 in the bone marrow and the use of steroids are related to immediate post-HSCT bone resorption that can lead to rapid bone mineral loss within the first few months after HSCT [18, 19]. Therefore, guidelines from the Center for International Blood and Marrow Transplant Research, the American Society for Blood and Marrow Transplantation, and European Group for Blood and Marrow Transplantation recommend screening via dual photon densitometry at 1 year after HSCT in adult women and in any patient who has received prolonged treatment with corticosteroids or calcineurin inhibitors [20] for early diagnosis and prevention of osteoporosis.

To date, several antiosteoporotic drugs have been used for treatment. At present, BPs are the most frequently prescribed drugs for HSCT-associated BMD loss [21]. Parathyroid hormone (PTH) and parathyroid hormone-related peptide (PTHrP) are generally avoided in patients who have undergone radiotherapy for the skeleton due to the association of teriparatide with osteosarcomas in animal models [22, 23]. Therefore, no clinical trials have been conducted to evaluate the effect of PTH or PTHrP in patients who have undergone HSCT. Selective oestrogen receptor modulators can decrease the risk of vertebral fractures. However, data regarding their potential to decrease nonvertebral fractures are insufficient [24]. In contrast, administration of denosumab, a human monoclonal antibody agent that inhibits receptor activator of nuclear factor-kappa B ligand (RANKL), is an effective antiresorptive therapy for osteoporosis that significantly reduces the incidence of vertebral and nonvertebral fractures. Bone loss after HSCT occurs predominantly in the cortical bone [25]. Our previous study indicated that after 1 year of HSCT, the decrease in femoral BMD (6.2%) was significantly greater than the decrease in lumbar BMD (2.2%) [26]. Due to the superior effect of denosumab on cortical bone compared to the effect of BPs [27] and its anti-osteoclastic activity, denosumab could be used as a promising treatment for HSCT patients. In addition, denosumab does not rely on renal clearance for metabolism or excretion and can be used without dose adjustment in patients with severe renal impairment [28]. Since some of the patients who have undergone successful transplantation exhibit impaired kidney function, the advantage of denosumab seems to be crucial for HSCT patients. However, the lack of data regarding its efficacy and safety has prevented its widespread use in HSCT patients.

In a prospective study that analysed the effect of denosumab at 1 year after kidney transplantation, denosumab induced an increase in BMD by 4.6% in the lumbar spine and by 2.3% in the total hip [29]. These results appear to be consistent with the results of the present study, which showed BMD increases of 4.39% and 1.80% in the lumbar spine and in the total hip, respectively. Since most of the HSCT patients had previously undergone cumulative steroid or immunosuppressive therapy with the development of GVHD, the effect of denosumab appears to be significant in HSCT patients. Several studies have reported that denosumab improved the strength of trabecular as well as cortical bone compartments [30–32]. However, the increase in lumbar spine BMD was greater than that in the femoral neck and total hip BMDs. Trabecular bone and cortical bone have different bone remodelling levels. Trabecular bone has a low matrix volume and a large surface area, whereas cortical bone has a large matrix volume and a small surface area. Therefore, the low surface area-to-volume ratio of the cortical bone leads to the lower accessibility of antiosteoporotic drugs inhibiting remodelling when compared with the trabecular bone [33]. Furthermore, the recovery of femoral BMD appears to be less than that of lumbar BMD, since bone loss is higher in the femoral neck than in the lumbar spine in HSCT patients, which might hinder recovery during the post-HSCT period [34–36]. Higher bone loss in the femoral neck might be due to the differences in tissue expression of several proteins such as bone morphogenetic protein 2, several growth factors, and their receptors related to bone metabolism [16].

A limitation of denosumab in HSCT patients is that it could increase the risk of infection by inhibiting the receptor activator of nuclear factor-kappa B [37, 38]. Severe adverse events such as cellulitis and erysipelas, which resulted in hospitalisation, occurred frequently in patients after receiving denosumab treatment in the Fracture Reduction Evaluation of Denosumab in Osteoporosis Every 6 Months (FREEDOM) trial [6]. A previous study has indicated that RANKL may be clinically useful to improve T-cell function recovery by controlling thymic regeneration in patients after HSCT [39]. Similarly, some of the patients in our study suffered from infections such as herpes zoster, pneumonia, and sinusitis during denosumab therapy. However, there was no clear clinical pattern suggesting an association of these infections with denosumab exposure. Discussions regarding denosumab-associated increase in infections might need to consider the fact that these patients were undergoing immunosuppressive therapy after transplantation. In addition, if denosumab has an immunomodulatory effect, it might also alleviate GVHD [18]. Controlled clinical trials are needed to evaluate the effect of denosumab on the immune system.

Our study has several limitations. The number of samples was insufficient to obtain significant statistical differences. Since our study used retrospective data, we could not fully reflect the effects of steroids, immunosuppressants, or whole body radiation therapy before or after HSCT. To evaluate the sole effect of denosumab quantitatively, a randomised control trial is needed in the future. In addition, the study period was too short to observe the occurrence of side effects of denosumab administration such as osteonecrosis of the jaw or atypical femoral fractures.

To the best of our knowledge, this is the first study showing the effects of denosumab in HSCT patients. Denosumab was well tolerated in HSCT recipients, and it improved their BMD. The use of denosumab could be a good therapeutic option without causing severe adverse effects in the recipients of haematopoietic transplantation. Follow-up studies on denosumab need to be conducted to evaluate its long-term effects on BMD and its safety regarding transplantation outcome.

## Figures and Tables

**Table 1:** Baseline clinical characteristics of the study population (*n* = 33).

Age (years)	52.6 ± 9.8
BMI (kg/m^2^)	21.4 ± 3.6
CTx (ng/mL)	0.62 ± 0.10
Serum calcium (mg/dL)	9.1 ± 0.7
Serum phosphorous (mg/dL)	3.4 ± 0.5
25-Hydroxyvitamin *D* (ng/mL)	30.3 ± 10.0
Type of malignancy, *n* (%)	
Acute myeloid leukemia	11 (33.3)
Acute lymphoid leukemia	14 (42.4)
Myelodysplastic syndrome	8 (24.3)
Baseline BMD (g/cm^2^)	
Lumbar spine	0.923 ± 0.143
Femur neck	0.723 ± 0.093
Total hip	0.728 ± 0.105
Steroid exposure, *n* (%)	22 (66.7%)

Continuous variables are presented as mean ± standard variation; categorical variables are presented as number (percentage); CTx, cross-linked C-terminal telopeptide of type 1 collagen; BMD, bone mineral density. Serum calcium level is adjusted calcium for albumin.

**Table 2:** Changes in biochemical markers during the study period.

	Baseline	6 months	12 months	18 months^a^	Percentage change^b^	*p* value
CTx (ng/mL)	0.62 ± 0.21	0.23 ± 0.17^*∗*^	0.22 ± 0.12^*∗*^	0.30 ± 0.18^*∗*^	−51.6%	<0.001
Calcium (mg/dL)	9.1 ± 0.7	9.0 ± 0.6	9.1 ± 0.4	9.0 ± 0.8	−1.1%	0.542
Phosphorous (mg/dL)	3.4 ± 0.5	3.5 ± 0.6	3.4 ± 0.4	3.5 ± 0.6	2.9%	0.381
25(OH)D (ng/mL)	30.3 ± 10.0	32.4 ± 11.1	32.3 ± 11.1	31.4 ± 11.3	3.6%	0.494

CTx, cross-linked C-terminal telopeptide of type 1 collagen; 25(OH)D, 25-hydroxyvitamin *D*; ^a^measured 6 months after the total of 3 denosumab injections administered at 6-month intervals; ^b^percentage change of baseline versus 18 months; *p* value by the one-way ANOVA and post-hoc analysis; Dunnett's method was applied for the post-hoc analysis; ^*∗*^*p* < 0.05 compared with the baseline.

## Data Availability

The data sets used and analysed during the current study could be made available upon reasonable request to the corresponding author.
